# Agonic Aspiration of Blood: Not Useful as an Animal-Based Indicator of Electrical Stunning Ineffectiveness in Pigs (*Sus scrofa domesticus*)

**DOI:** 10.3390/ani13142292

**Published:** 2023-07-13

**Authors:** Maria Francisca Ferreira, Emma Fàbrega, Isabel Pires, Maria Madalena Vieira-Pinto

**Affiliations:** 1 School of Agrarian and Veterinary Sciences (ECAV), University of Trás-os-Montes and Alto Douro (UTAD), 5001-801 Vila Real, Portugal; 2 Animal Welfare Program, IRTA, 17121 Monells, Spain; emma.fabrega@irta.cat; 3 Department of Veterinary Sciences, University of Trás-os-Montes and Alto Douro (UTAD), 5001-801 Vila Real, Portugal; ipires@utad.pt (I.P.); mmvpinto@utad.pt (M.M.V.-P.); 4 CECAV—Veterinary and Animal Research Centre, University of Trás-os-Montes and Alto Douro (UTAD), 5001-801 Vila Real, Portugal; 5 Associate Laboratory for Animal and Veterinary Sciences (AL4AnimalS), 5001-801 Vila Real, Portugal

**Keywords:** agonic aspiration of blood, animal welfare, electrical stunning, horizontal bleeding, pigs, slaughter

## Abstract

**Simple Summary:**

Agonic aspiration of blood should be seen as a critical welfare problem in the slaughter of pigs, as it reflects a moment of pain, distress, and fear for the animal during its time of death. Our survey focused on assessing whether lesions of agonic blood aspiration could be used as a valuable indicator to guide the official veterinarian to immediately apply the necessary measures to ensure good practices of animal welfare at slaughter. Ineffective electrical stunning of pigs has been associated with the incidence of lung lesions suggestive of agonic blood aspiration at postmortem inspection. However, for the first time, the findings from this study indicate that aspiration of blood may be related to factors other than agonic status during slaughter process. One of them being that horizontal bleeding may favour slaughter technopathies. This process should not be neglected, as it may result in lesions resembling those resulting from the agonic lung aspiration of blood—which is not related to an agonic status.

**Abstract:**

Agonic aspiration of blood (AAB) may result from an inadequate exsanguination with accidental trachea severing, that can be favoured by ineffective stunning of pigs (*Sus scrofa domesticus*). This study aimed to evaluate AAB as an animal-based indicator of electrical stunning ineffectiveness, which could be used by official veterinarians during the post-mortem inspection of pigs. Information on 3584 finishing pigs was collected at a Portuguese abattoir that performs head-to-body electrical stunning with horizontal bleeding. Of them, 15.5% of the pigs presented signs of ineffective stunning. AAB lung lesions were found in 27.8% of lungs. Despite what was predicted, a strong correlation was found between well stunned animals and the presence of blood lesions in lungs (*p* = 0.006). Statistical significances were found between pigs’ lateral recumbency at the conveyor and the presence of blood affecting one lung. Under the conditions of this study, the authors cannot point to AAB lung lesions as an animal-based indicator of electrical stunning ineffectiveness. Further studies should be conducted to establish a better understanding of the causes of aspiration of blood, in particular how horizontal bleeding may affect the occurrence of similar lesions.

## 1. Introduction

Animal welfare has become an emerging topic in the European Union (EU) in the past 40 years [1,2].

According to regulation, animals are considered to be sentient beings and hence slaughter procedures such as killing and related operations should spare any avoidable pain, distress or suffering [3]. Stunning animals before killing is compulsory (except for the production of meat in a context of ritual slaughter) with the purpose of intentionally causing loss of consciousness and sensibility without pain, until death is confirmed after bleeding [3]. Unconsciousness can be achieved by inducing a reversible or irreversible dysfunction of brain structures such as the cerebral hemispheres, the reticular formation or the ascending reticular activating system [4]. Currently, electrical stunning and exposure to carbon dioxide are both common methods for stunning swine [5]. The latter might result in inferior quality carcasses with a higher incidence of bruising, haemorrhages and bone fractures, when animals are exposed to a poor handling and an incorrect performance [5]. This emphasizes the need for animal welfare legislation at the abattoir to ensure that slaughter and killing operations are performed properly—in order to minimise animal suffering and, consequently, improve the quality of the final product.

When applying electrical stunning, pigs can recover consciousness within about 30 to 60 s after the stun [6]. Thus, the European Food Safety Authority Panel’s on Animal Health and Welfare (EFSA-AHAW) has reviewed animal-based measures (ABMs) to assess the state of consciousness after stunning, during sticking and during bleeding. Examples of those are rhythmic breathing, corneal reflex, righting reflex and voluntary vocalizations [7].

Correctly performed head-to-body electrical stunning also impairs cardiac output (reduced to less than 30% of normal) and normal blood circulation with consequent hypoxia in the brain and myocardium [8,9]. This can be done by using a single current cycle, in which electrodes are placed on either side of the head to induce unconsciousness. A third electrode is placed on the chest to induce cardiac ventricular fibrillation, and thereby could cause the death of the animal through cardiac arrest [7].

A conscious pig, or a pig regaining consciousness at bleeding, may perform more body movements that can lead to an incorrect sticking technique with severed trachea along with the brachiocephalic trunk [10]. Consequently, blood can be aspirated into the pigs’ lungs and the animal can suffer because the sensory receptors of the respiratory tract will respond to inhaled threats, leading to a cough reflex or an expulsion reflex [11,12]. For this reason, lung lesions related to blood aspiration are commonly referred to Agonic Aspiration of Blood (AAB) [13,14]. At post-mortem inspection, AAB lesions can be detected at the lungs, characterized by punctiform haemorrhage pattern or with a lobular delimitation (known as chessboards spots) [15].

This gross non-pathological finding may be considered poor welfare, associated with pain and suffocation, and resulting from a conscious animal inhaling blood during the bleeding process [8]. For that reason, it could help the official veterinarian to determine that the stunning method is not being effective, and also that the sticking technique is not being well performed. However, is this true under all circumstances? To evaluate this hypothesis this study was performed, having as main objectives to:

(i) evaluate the grade of ineffectively electrically stunned pigs during bleeding; (ii) evaluate the incidence of lung lesions suggestive of agonic blood aspiration, according to their extent at post-mortem inspection; and (iii) analyse the association of electrical stunning ineffectiveness with lung lesions suggestive of agonic blood aspiration in slaughtered pigs, in order to determine if agonic blood aspiration in pig’s lungs can be used as an animal-based indicator of electrical stunning ineffectiveness.

## 2. Materials and Methods

A prospective, observational study was performed including 73 batches of finishing pigs (*Sus scrofa domesticus;* pigs with 80–100 kg live weight from the same genetic line Large White x Landrace) from 23 conventional indoor herds randomly assigned in a commercial abattoir for domestic ungulates, located in the Region of Lisbon, Portugal, during 4 consecutive weeks. For that reason, no ethical approval was required. Animals were transported to the abattoir in accordance with the European legislation [16] and submitted to the same handling once at the slaughterhouse.

Batches were also randomly assessed. The assessment started at the beginning of slaughter and finished with the last batch slaughtered before lunch time. A batch was defined as all pigs from one herd sent for slaughter on the same day. The evaluation of each batch always started when the first animal was slaughtered.

Data collection was conducted at different stages of observation in the abattoir, namely at the bleeding platform and at post-mortem inspection (PMI). All assessments, post-stunning and post-mortem inspection, were always performed by the same individual of the research team. For that reason, the sample size by batch was estimated based on the time it took the same assessor to evaluate the pigs during bleeding and reach the post-mortem inspection site for lung evaluation.

The abattoir used a restrainer-conveyor (“Midas Restrainer”) where pigs were first restricted to a single-line raceway. The system led and immobilized the pigs to a head-to-body electrical stunning system with an automatic electrode placement. The stun was performed by applying a constant amperage (1.3 A) for 3–4 s. This equipment operated with higher voltage working at outputs in excess of 200 V. After stunning, the animals fell onto a table where initial bleeding took place. In the present study, sticking was considered to begin from the moment that a knife was pushed into the chest of the animal, which signalled the end of the stun-to-stick interval. This interval was measured with a chronometer during a day of slaughter at different times of day.

Afterwards, animals were hoisted and kept bleeding for an average of 15 min before reaching the vertical scald. After going through the vertical scald, carcasses were submitted to automatic dehairing. At this stage, carcasses were mixed for a small period of time (6 to 8 min), which accounted for the impossibility to establish an equal order between pigs seen during bleeding and their carcasses presented for post-mortem inspection. Unfortunately, it was not possible to find any method that could withstand the slaughtering procedures (namely scalding, dehairing and singeing).

### 2.1. Data Collected at Bleeding Platform

Pigs were continuously observed for physical signs that could indicate consciousness or the risk of imminent recovery. Hence, five animal-based measures (ABMs) were selected and recorded in each pig for individual assessment, according to the scientific opinion of [17]: rhythmic breathing, righting reflex, corneal reflex, spontaneous blinking and voluntary vocalizations (Table 1). The assessor was positioned just after sticking and could observe the animals during the first 2/3 of bleeding for assessment.

The frequency of ABMs shown individually or in combination was evaluated. To assist with the practical assessments, a stun-ineffectiveness score (SIS) was designed to categorize ABMs signifying risk of consciousness recovery as follows (adopted from [18]):

- Grade 0: Pigs were considered adequately stunned if they were in a position of whole-body relaxation and there was no evidence of rhythmic breathing and righting reflex.
- Grade 1: Pigs with a display of rhythmic breathing and/or righting reflex were considered inadequately stunned at a high risk of poor animal welfare.
- Grade 2: Pigs with a display of rhythmic breathing and/or righting reflex with additional presence of corneal reflex, spontaneous blinking and/or voluntary vocalizations were considered inadequately stunned and at the highest risk of poor animal welfare.

All animals displaying rhythmic breathing and/or righting reflex were identified by the slaughterman staff as not being properly stunned. In these circumstances, an effective measure was applied by repeating the application of electrical stunning.

Additionally, the lateral recumbency to which the pig was laying down during sticking and initial bleeding (right or left) was also evaluated.

### 2.2. Identification of Lesions Suggestive of Agonic Blood Aspiration

Lungs were identified as having lesions suggestive of AAB when blood was visible on the parenchyma as a haemorrhagic punctiform pattern, a mosaic pattern (lobular delimitation) or with a diffuse pattern (an extension of the previous one). The edges of the lungs were cut to confirm the presence of blood inside the lumens of large bronchi and bronchioles.

Each lung was evaluated based on gross morphologic changes (mainly distribution of lesions, texture of lung by palpation and presence of grossly visible exudate) to discard differential diagnosis. Presence of cranioventral consolidation with slightly depressed dark-red lobules and a firm texture by palpation were features used as indicative of enzootic pneumonia.

The presence of focal protruding nodules associated with fibrinosuppurative, haemorrhagic or necrotizing lobar pneumonia was described as typical lesions of *Actinobacillus pleuropneumoniae*. The presence of abscesses (if chronic) or presence of randomly scattered foci of hyperaemia/haemorrhage were features used as indicative of embolic pneumonia. Interstitial pneumonia was characterized by patchy, lobular or diffuse distribution of colour variation (from red to a pale whitish colour) and changes in consistency (firm texture) [19,20].

### 2.3. Extent of Lesions Suggestive of Blood Aspiration

**Figure 1:**
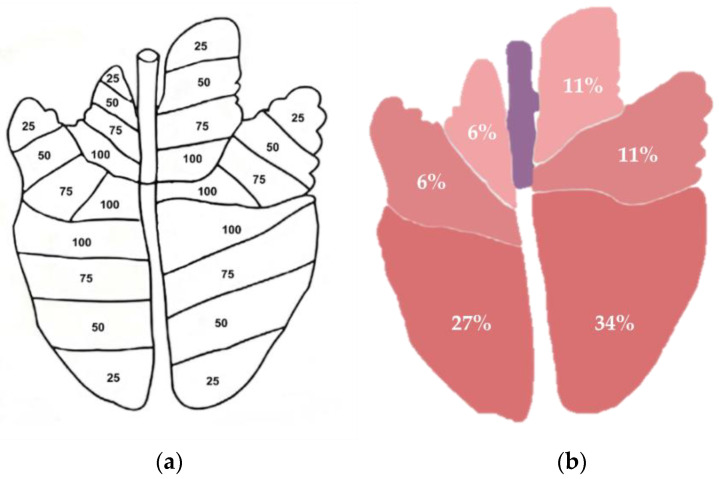
Schematic representation of a pig lung. Dorsal view. (**a**)—Divided by lobes and each of the lobe divided by four categories of percentage: 25, 50, 75 and 100. Adapted from [13]. (**b**)—Lobes relative sizes of a pig lung. Adapted from [21]. The accessory lobe was not evaluated.

A classification was applied based on the pulmonary lobes of the pig (cranial, middle, and caudal lobes for right and left lungs). The accessory lobe was not considered since the lung was in dorsal position and there was no direct vision of this structure. To facilitate data collection at the abattoir, the diagram shown in Figure 1a was applied. For each lobe, five categories of percentages were defined, based on the study of [13]: (0) absence of blood at lobe surface, (25) blood representing less than 25% of the lobe surface; (50) blood representing 26% to 50% of the lobe surface; (75) blood representing 51 to 75% of the lobe surface; and (100) blood representing more than 75% of the lobe surface.

Afterwards, the extent of the agonic blood aspiration was re-calculated by means of a two-dimensional approach, where the affected lobe surface was corrected by its relative size (Figure 1b). The weight of each pulmonary lobe was assigned according to [21]. The area of extent of the total lung surface was calculated based on the sum of the six lobes percentages. The lung was scored as 0 when no blood was present. Scores 1 to 4 indicated the presence of lesions suggestive of AAB, as follows: grade 1 for lesions affecting 1–25% of the lung; grade 2 for lesions affecting 26–50% of the lung; grade 3 for lesions affecting 51–75% and grade 4 for lesions affecting 76–95% of the lung. The total area of the affected lung could not reach above 95%, since the score system includes the accessory lobe (relative size of 5%) that was not evaluated in the present study.

### 2.4. Histopathological Analysis

To confirm the diagnosis of lesions of AAB, samples from 22 lungs displaying different extent grades were randomly selected from different batches under analysis. The samples were placed in an individual plastic container with 10% formaldehyde and stored away from light and heat, for further processing at the Laboratory of Histology and Veterinary Pathological Anatomy of UTAD, Vila Real, Portugal.

The lungs were placed dorsal surface uppermost on a clean table for photograph report and a portion of the lungs were incised with a blade for further histopathologic analysis. Examples of these lungs and their photograph registration are provided in the Supplementary Materials.

The histopathological evaluation characterized each sample based on the presence of erythrocytes in the sites of gaseous exchange (alveoli, alveolar sac, alveolar ducts and respiratory bronchiole), inside of bronchiole, or inside of small and large bronchi.

All samples had the presence of erythrocytes in the three criteria used to assess the microscopic analyses. Despite the inner of the trachea was not evaluated during the assessment phase, our histopathological findings suggest the presence of blood in the lower respiratory tract.

Moreover, no pathological process was identified in an extent grade worth noting. These results allowed us to classify with certainty the extent grades used for the lungs assessments.

**Figure 2:**
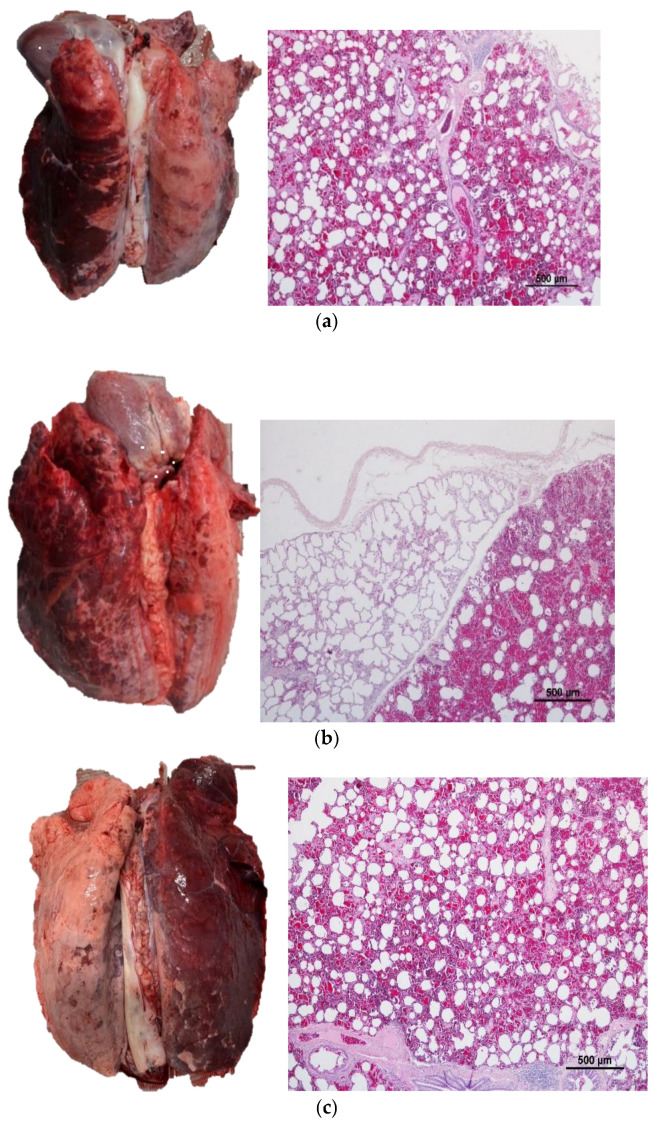
Macroscopic image of pig lungs and respective microscopic findings. (**a**)—Predominance of erythrocytes in mostly all alveoli, alveolar sac, alveolar ducts, and bronchiole. Sample taken on the left lung. (**b**)—Presence of two lobules separated by an interlobular septum. The right one had erythrocytes in most of the alveoli, alveolar sac and alveolar ducts, and small bronchiole. Note that normal alveolar spaces were rarely observed throughout the pulmonary parenchyma, whereas the left lobule had practically no presence of erythrocytes. (**c**)—Predominance of erythrocytes in mostly all alveoli, alveolar sac, alveolar ducts and bronchiole. Sample taken on the right lung. Hematoxylin and eosin.

Figure 2a–c are examples observed during the study and their respective histopathologic analysis.

### 2.5. Statistical Data Analysis

Registration and organization of data were performed using the program Microsoft^®^ Excel 2016. Data was divided in accordance with the different states of assessment: data of the animals seen at the time of bleeding; and data concerning lung analysis. Additionally, the data collected was introduced at an individual level (by animal and by lung).

Statistical analysis was performed using SPSS Statistics for MacOS, version 26.0 (SPSS, Chicago, IL, USA). Continuous variables were described in median and interquartile range (IQR). Categorical variables were described using absolute and relative frequencies. Univariate analysis was first performed using non-parametric tests, including the Mann–Whitney test and Spearman’s correlation coefficient. The relation between two categorical variables was described using the Chi-square test (Fisher’s exact test was used whenever indicated). Odds ratio was calculated to evaluate the magnitude and direction of any associations performed at a batch level. Statistical significance was set up at *p* < 0.05.

## 3. Results

A total of 73 batches from 23 herds were included in the study, corresponding to a population of 11,152 animals. Four animals (0.03%) died during transport or on arrival and another four (0.03%) died during lairage, failing to follow the normal slaughter procedures and were, therefore, excluded from this study, resulting in a final total of 11,144 finishing pigs.

Out of the 11,144 finishing pigs, 3584 pigs and their respective lungs were analyzed at an individual level for the study, with a mean of 49 animals per batch (range: 28–50). Regarding lung analysis, 22 lungs were discarded from the study due to chronic pleural lesions. Another 9 lungs were removed from analysis due to data collecting gaps, resulting in a final sample of 3553 lungs for statistical data analysis.

### 3.1. ABMs and Stun-Ineffectiveness Score

A breakdown of the stun-ineffectiveness score (SIS) by grades and the prevalence of the different ABMs in each group is represented in Table 2. Most of the pigs (3028; 84.5%) were well stunned. From the remaining pigs, 410 pigs (11.4%) were classified as grade 1 and 146 pigs (4.1%) as grade 2, with a total of 556 pigs (15.5%) presenting ABMs of inadequate stunning.

ABMs evaluation at the bleeding platform revealed that rhythmic breathing was present in 551 pigs (15.4%), corneal reflex in 290 pigs (9%), spontaneous blinking in 74 pigs (2.1%), righting reflex in 10 pigs (0.3%) and voluntary vocalizations in 2 pigs (0.1%) (Table 2). In all animals, such indicators appeared at variable times during the first 2/3 of the bleeding period.

A statistically significant association was found between the presence of rhythmic breathing and the remaining ABMs (Table 3). From the 551 pigs with rhythmic breathing, 98 pigs showed simultaneously corneal reflex, 46 spontaneous blinking and 2 animals vocalizations.

No statistically significant association was found between the righting reflex and the other ABMs, except with rhythmic breathing, with an odds ratio (OR) of 5.55 [95% confidence interval (CI): 1.60–19.22; *p* = 0.002] (Table 3). Associations among the remaining ABMs were not studied since they are considered less reliable if presented independently or if missing a more consistent indicator, such as rhythmic breathing and righting reflex (EFSA-AHAW 2013).

### 3.2. Lung Evaluation: AAB Lesions

At post-mortem lung evaluation, lesions suggestive of agonic aspiration of blood were detected in a total of 989 lungs (27.8%). After applying the scoring system based on the lesions’ extent, 514 lungs (14.5%) were classified as grade 1, 369 lungs (10.4%) as grade 2, 94 lungs (2.6%) as grade 3 and 12 lungs (0.3%) as grade 4.

### 3.3. Relationship between the Stun-Ineffectiveness Score and AAB Lesions in the Lungs

Table 4 presents the correlation between the SIS and the presence of lesions of blood in the lungs at a batch-level analysis. Despite what was predicted to be found, the correlation of the mean number of animals poorly stunned (grade 1 and 2 of the SIS) and the mean number of AAB lesions in the lungs (grades from 1 to 4 of the extent score) did not reach any statistical significance.

On the other hand, a strong correlation between the mean number of properly stunned animals and the mean number of AAB lesions present in lungs was found at a 0.01 level. The coefficients are positive and high, showing that the higher the mean number of properly stunned pigs, the higher mean number of lungs with presence of AAB lesions (Table 4).

### 3.4. Relationship between Lateral Recumbency at the Conveyor and Presence of Blood in the Lungs

A total of 1822 pigs fell on their right lateral recumbency (50.8%) and 1762 pigs fell on their left lateral recumbency (49.2%) after the electrical stunning. These results suggest the pig’s position at the platform for sticking and initial bleeding to be random.

We further analyzed, for each set of lungs, which side was predominantly affected by the presence of blood (right or left lung; Table 5). More than 44% of lungs were affected exclusively on their right lung and more than 25% of lungs were affected exclusively on their left lung (with a total of 69.8% lungs), against 30.2% lungs affecting both lungs.

**Figure 3:**
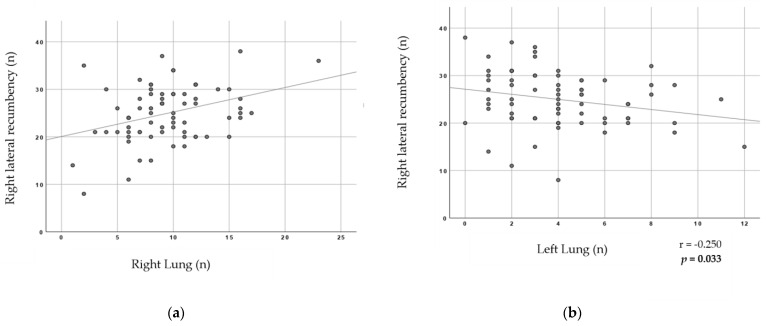
Scatter Graphs for pig’s lateral recumbency on their right side and presence of AAB lesions in lungs variables at a batch-level analysis. Spearman’s correlation coefficients and respective *p* values are shown in the lower right side of each graph. The significant level of probability is highlighted in bold. (**a**) Right lateral recumbency of pig according to AAB lesions on the right lung. (**b**) Right lateral recumbency of pig according to AAB lesions on the left lung.

**Figure 4:**
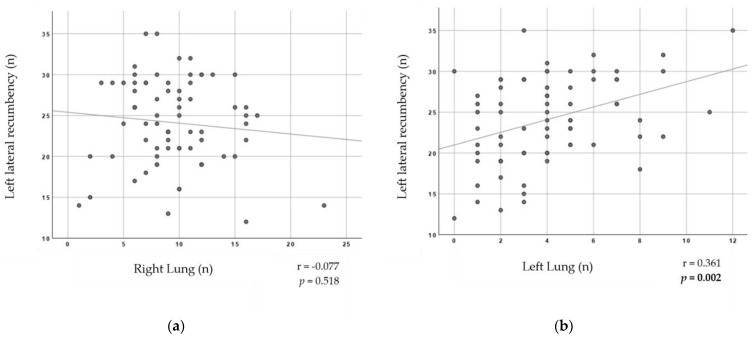
Scatter Graphs for pig’s lateral recumbency on their left side and presence of AAB lesions in lungs variables at a batch-level analysis. Spearman’s correlation coefficients and respective *p* values are shown in the lower right side of each graph. The significant level of probability is highlighted in bold. (**a**) Left lateral recumbency of pig laying down according to AAB lesions on the right lung. (**b**) Left lateral recumbency of pig laying down according to AAB lesions on the left lung.

Statistical significances were found between the pig’s lateral recumbencies at the conveyor (left or right lateral recumbency) and the presence of blood lesions affecting exclusively or mostly one lung (left or right) at a batch-level analyses (Figure 3 and Figure 4). A positive correlation between the mean number of pigs falling on their right lateral recumbency and the mean number of lungs with predominance of blood on the right lung is represented in Figure 3a (r = 0.264; *p* = 0.024). Additionally, the higher mean number of pigs falling on their right lateral recumbency, the lower mean number of lungs with predominance of blood on the left lung (Figure 3b; r = −0.250; *p* = 0.033).

The same can be said about pigs falling on their left lateral recumbency. The higher of mean number of pigs falling on their left lateral recumbency at the conveyor, the higher the mean number of lungs with predominance of blood on their left lung (Figure 4b; r = 0.361; *p* = 0.002).

Pigs lying on their left lateral recumbency had lower association with the mean number of right lungs with blood lesions, although it did not reach any statistical significance (Figure 4a; r = −0.077, *p* = 0.518).

## 4. Discussion

The assessment of stun-ineffectiveness in pigs after head-to-body electrical stunning can be performed at three key stages: after stunning, during sticking and during bleeding [17]. For our survey, we selected the latter stage to analyse welfare indicators in pigs, since the narrow space of the stunning-sticking area and the short stun-to-stick interval did not allow stun-ineffectiveness indicators to be observed after the release of pigs from the stun box. Nevertheless, the assessor could see the animals from the moment of stunning until they reached the bleeding platform. According to [22] for pigs, the indicators should be individually assessed, whenever possible, from the moment of stunning until 1 min after sticking.

Rhythmic breathing was the most frequent ABM observed in pigs at bleeding, underpinning the results from [23]. One interesting result is the low number of pigs with attempts to regain posture (arching the neck or the body) against the number of pigs with resumption of breathing. A possible cause for this is the use of a lower current, inducing an electro-immobilization which, despite the consciousness state of the pig, can result in immediate loss of posture [17]. Nevertheless, our results regarding these two ABMs are in accordance with the ones found by [24], in which 80% of the pigs not properly stunned had rhythmic breathing against 5% attempts to raise.

Monitoring of stunning should always involve the evaluation of various indicators to effectively recognize outcomes of consciousness [4]. Head-to-body stun extends the period of unconsciousness or leads to immediate death of the animal, meaning that the probability of an animal showing any signs of consciousness is seriously diminished [8,9]. However, our results suggest that 15.5% of the pigs evaluated were regaining consciousness or were already conscious at the time of assessment, which is remarkable and an obvious cause for concern from an animal welfare point of view. [7] has described several reasons that can lead to failure in the onset of unconsciousness at this stage, namely the wrong placement of the electrodes, lack of good electrical contact, application of too low currents and lack of calibration of the equipment. The majority of them are technical problems that affect the quality of stunning, compromising animal welfare [25]. Poorly stunned animals may develop primary and secondary hyperalgesia, which is characterized by an increase in pain perception and sensitivity with prolonged response to harmful stimulus [25]. To avoid this phenomenon, it is critical to frequently check for signs of recovering consciousness to immediately re-stun the animals.

After analysing our results, we suggest the SIS or other similar approaches [18,26] as a practical tool to monitor the risk of inferior animal welfare following stunning at slaughter. We suggest that the presence of rhythmic breathing during bleeding indicates the imminence of regaining consciousness. The presence of this indicator with the remaining ABMs confirms that the animal is conscious, revealing the highest risk to animal welfare. Any animal scored with grade 1 or 2 of the SIS at bleeding should be re-stun to reduce the duration of spontaneous physical activity [27].

Inadequate sticking constitutes a potential welfare issue when it is delayed (related to the stun-to-stick interval) or is not properly done with bleeding being too slow (related to incomplete severance of the main blood vessels) [28]. To discard the first cause, stun-to-stick interval was measured with a chronometer during a day of slaughter at different hours, which gave a mean of 6 ± 1.79 s. Even though stun-to-stick interval is in principle not critical under this electrical stunning method [8], this criterion can only be applied if in fact there is always a correct stun involving the heart cycle. Nevertheless, this value is within the one recommended (maximum acceptable is determined as 15 s after stunning [6]. Even though our survey cannot establish whether there were errors regarding the sticking technique, a poorly performed technique, along with failure of the electrical stun, can lead to cerebral blood supply with prolonged brain activity—leading to a higher risk of the animal regaining consciousness during bleeding [7].

Blood lung lesions are widely accepted as a possible consequence of blood aspiration when the trachea is accidentally cut at bleeding [19,20]. If the animal does not lose consciousness rapidly whilst blood is present in the respiratory tract, receptors in the airways will make them try to cough or have an expulsion reflex, thus suffering may be noticed in a pig under these circumstances [10,12]. This can only happen if the vagus nerve is maintained intact after cutting, otherwise animals may not show any signs of fear and pain [11].

However, few to no surveys were found regarding this phenomenon [10]. With the aim of finding valuable causes for the results presented, studies from cattle slaughter without stunning (e.g., religious slaughter) are going to be used as supporting scientific evidence. In this case, the carotid arteries, jugular veins, trachea and oesophagus are severed in a single motion on a fully conscious animal [29].

A study in cattle slaughtered without pre-stunning showed that they had high percentages of blood lining in the respiratory tract and the upper bronchi [30]. This result is similar to [14]’s and to [11]’s surveys. Two arguments were proposed by [30] to justify the presence of blood at the respiratory tract: either blood was aspirated with air through the severed trachea; or the trachea was not cut but rupture of the alveolar-capillary barrier occurred due to negative airway pressures. The work by [14] revealed the first argument as the main cause: most of the animals from the Shechita group, a ritual slaughter, had agonal respiration and frequent cut of the end of the trachea was observed.

These studies are important to confirm that blood lining in the respiratory tract can be highly associated with breathing actions. [13], in their book about meat inspection in pigs, stated that the “repeated evidence of agonic aspiration lesions in the lung at the time of post-mortem inspection should alert the OV to the possibility of occurrence of indirect failure in the stunning process and, consequently, in the welfare of the animals”.

So, there was a logical point in linking the stun-ineffectiveness score with the presence of blood lung lesions. However, our statistical results (*p* = 0.006) indicate that the higher the mean number of properly stunned pigs, the higher the mean number of lungs with presence of blood. Nevertheless, it is important to note that, in our study, the cut of trachea was not evaluated, which limits the explanation of the results. The observation of the inner of the trachea could have supported aspiration of blood [30]. Unfortunately, this was not possible to assess because the high-speed rate of the line made it unmanageable to perform this observation. Furthermore, it was noticed several times that the trachea was accidentally cut by the operators when separating the viscera for post-mortem inspection, which would have led to unreliable results. However, a recent survey performed in Colombia concluded that abattoirs performing horizontal bleeding had a higher percentage of cutting trachea than abattoirs performing vertical bleeding [31].

Regarding the extent of blood present at the lung examination, two explanations are proposed given the amount of blood entering the respiratory tract: either blood can dispersedly reach the alveoli in fine particles as a result of forced inspiration (leading to the presence of punctiform petechias), or a higher amount of blood can reach the trachea and enter bronchiolar branches (leading to lesions with a polygonal delimitation) [32,33]. This could explain the predominance of grades 1 and 2 of the extent score in the present study. However, the authors did not find any literature that could explain the presence of a severe amount of blood in the lung’s surface (grades 3 and 4 of the extent score).

AAB lesions are normally visible on both the pleural and the cut surfaces of the lungs [20]. In our study, lesions were frequently seen on only one lung, corresponding to the pig’s lateral recumbency at the conveyor (left or right side). For that reason, the authors believe that horizontal bleeding may have a negative impact on the lung blood circulation at exsanguination, thus lesions resembling AAB lesions in the lung may appear. In this case, the ventricular fibrillation of the heart caused by the electric stunning system would impair venous reflux on the veins, resulting in added pressures on the lung that is underneath on the conveyor, with consequent rupture of the alveolar-capillary vessels.

Although AAB lesions could be confused with hypostasis lesions, our histopathological findings showed an enhanced presence of erythrocytes in the sites of gaseous exchange (alveoli, alveolar sac, alveolar ducts and respiratory bronchiole), inside of bronchiole, or inside of small and large bronchi, supporting that blood was present in the respiratory tract, rather than congestion of blood on the pulmonary veins. It is also important to properly distinguish this non-pathological finding, which resembles pulmonary haemorrhage or infectious pneumonias, from hepatization areas, leading to an erroneous diagnosis [32]. In the case of aspiration of blood, the lung is not consistently modified at palpation and there are visible blood-filled lobules, demarcated by a thin and unaffected interlobular tissue, when section of the lung is performed [15].

Despite the fact that gasping should not be confused with rhythmic breathing, it is still described as intermittent and non-organized movements that can be present before onset of death [4]. In other words, gasping can be observed after a successful electrical stun [34]. This can, therefore, explain the results of our study regarding the presence of AAB lesions in both lungs. Even though no literature was found to support this hypothesis, an unconscious animal with simultaneously severed trachea and gasping may also inhale blood, which would result in the presence of lung lesions similar to the ones found when an agonic process occurs.

## 5. Conclusions

The findings revealed that, under the condition of this study, the official veterinarian cannot use agonic blood aspiration lesions in the lungs as an animal-based indicator of electrical stunning ineffectiveness. A strong correlation was found between well stunned animals and the presence of these lesions in the lungs; as well as statistical significances between pig’s lateral recumbency on the conveyor and the presence of blood lesions affecting exclusively or mostly one lung (right or left lung). The results from this study indicate, for the first time, that horizontal bleeding may have a negative impact on the lung blood circulation at exsanguination, thus lesions resembling AAB lesions in the lung may appear. This process should not be neglected, as it is not related to an agonic status.

Therefore, specially under the conditions of our study, agonic aspiration of blood is not a good predictor of animals poorly stunned.

Nevertheless, our results suggest that more than 15% of the pigs evaluated were regaining consciousness or were already conscious at the time of assessment, which is remarkable and an obvious cause for concern from an animal welfare point of view. We highly recommend the use of practical approaches to describe animal-based measures of inadequate stunning rated for risk of inferior animal welfare at slaughter. Any animal considered ineffectively stunned should receive the application of a second stun. Corrective measures should also be implemented at the abattoir to reduce the likelihood of failing electrical stuns, or even the adoption of other more effective stunning systems.

More research regarding this topic should be undertaken to stablish a better understanding of the mechanisms behind aspiration of blood and to which extent may affect animal welfare. More surveys are also required to understand the impact of horizontal bleeding on slaughter technopathies.

## Figures and Tables

**Table 1:** Animal-based measures (ABMs) for assessment of the state of consciousness in pigs after head-to-body electrical stunning during bleeding (Adapted from [14]).

ABMs	Definition
Rhythmic Breathing	Rhythmic air inhalation seen in the form of regular expansion/contraction of chest or flank area or feeling rhythmic air exhalations on the back of the hand.
Righting Reflex	Fail to collapse or arching of back in animal’s attempt to right itself or raising of the head.
Spontaneous Blinking	Animal blinks his eye on its own without stimulation.
Voluntary Vocalizations	Animal squeals or groans using vocal cords not associated with involuntary sounds during the dying process.
Corneal Reflex	Corneal reflex was tested by carefully touching the corneal area with a pen tip angled at approximately 45°. If the pig blinked in response it was noted as a corneal reflex.

**Table 2:** Prevalence of ABMs within grades of the stun-ineffectiveness score (SIS) on pigs examined individually at the bleeding platform of the abattoir.

		Animal-Based Measures (ABMs)				
		Rhythmic Breathing (*n* = 551; 15.4% ^1^)	Righting Reflex (*n* = 10; 0.3% ^1^)	Corneal Reflex (*n* = 290; 9% ^1^)	Spontaneous Blinking (*n* = 74; 2.1% ^1^)	Vocalizations (*n* = 2; 0.1% ^1^)
Stun-ineffectiveness score (SIS) *n* = 3584 pigs	Grade 0			192	28	0
	(*n* = 3028; 84.5% ^2^)			(6.3% ^3^)	(0.9% ^3^)	
	Grade 1	405	9			
	(*n* = 410; 11.4% ^2^)	(98.8% ^3^)	(2.2% ^3^)			
	Grade 2	146	1	98	46	2
	(*n* = 146; 4.1% ^2^)	(100% ^3^)	(0.7% ^3^)	(67.1% ^3^)	(31.5% ^3^)	(1.4% ^3^)

^1^ Percentage of prevalence of each ABM for *n* = 3584 pigs. ^2^ Percentage of prevalence of each grade score. ^3^ Percentage of prevalence of each ABM within each grade score.

**Table 3:** Association between rhythmic breathing and the remaining AMBs presented in pigs examined individually at the bleeding platform of the abattoir.

	Rhythmic Breathing			
	Pearson Chi-Square	*p* Value	Odds Ratios	
			Estimate	95% IC
Rhythmic Breathing	-	-	-	-
Righting Reflex	$x^{2}\left(1\right)=9241$	0.002	5.55	1.60–19.22
Spontaneous Blinking	$x^{2}\left(1\right)=127,139$	0.001	9.78	6.05–15.79
Corneal Reflex	$x^{2}\left(1\right)=8228$	0.001	3.20	2.46–4.16
Vocalizations	$x^{2}\left(1\right)=6072$	0.014	11.05	1.00–122.02

**Table 4:** Spearman correlation coefficients for lungs with AAB lesions and SIS grades at a batch-level analysis (*n* = 73 batches).

Variables	SIS		
	0	1	2
	(41.48; 25–50)	(5.62; 0–13)	(2.0; 0–6)
Lungs with AAB lesions	0.320	−0.107	0.017
	***p* = 0.006**	*p* = 0.358	*p* = 0.887
Right Lung	0.342	−0.075	−0.091
(24.96; 8–38)	***p* = 0.003**	*p* = 0.530	*p* = 0.046
Left Lung	0.053	−0.038	0.191
(24.14; 12–35)	*p* = 0.654	*p* = 0.750	*p* = 0.106

Mean, minimum and maximum for each variable are described within brackets. The significant level of probability is highlighted in bold.

**Table 5:** Prevalence of lung lesions suggestive of AAB for each side of the lungs.

	Lungs with Lesions of AAB (*n* = 989 Lungs)	
	Right Lung	Left Lung
Affecting exclusively or mostly one side of the lung	692	297
(*n* = 989 lungs)	(70% ^1^)	(30% ^1^)
Affecting exclusively one side of the lung	441	249
(*n* = 690 lungs)	(44.6% ^1^)	(25.2% ^1^)

^1^ Percentages of prevalence are reported within brackets.

## Data Availability

The data presented in this study are available on request from the corresponding author. The data are not publicly available due to privacy reasons.

## Supplementary Materials

The following supporting information can be downloaded at: https://www.mdpi.com/article/10.3390/ani13142292/s1, Figure S1: Atlas of lung lesions suggestive of agonic blood aspiration seen during post-mortem inspection.
