# Supplementary material for: Agonic Aspiration of Blood: Not Useful as an Animal-Based Indicator of Electrical Stunning Ineffectiveness in Pigs (Sus scrofa domesticus)
Source: Animals (Basel). 2023 Jul 13;13(14):2292. doi: 10.3390/ani13142292 (PMC10376572; doi:10.3390/ani13142292)
Supplement: Supplementary file 1 [file animals-13-02292-s001.zip › Figure S1.pdf]

Atlas of lung lesions suggestive of agonic blood aspiration seen during post mortem inspection

Note to the reader

- The photos are original.
- The photos were taken at a commercial horizontal abattoir for domestic ungulates, located in the Region of Lisbon, Portugal, between October and November 2021.
- Pigs' lungs suggesting agonic blood aspiration lesions at different extent grades were selected and taken from the slaughter procedures line at post mortem inspection site. The lungs were selected from 73 random batches of finishing pigs (*Sus domesticus*) from 23 conventional indoor herds randomly assigned to the study.
- Lungs may suggest concomitant pathological lesions based on gross morphologic changes.
- Each set of lungs was placed dorsal surface uppermost on a clean table for photograph report.
- A knife was used to perform a transversal cut of the caudal lobes and a portion of the lung site suggesting aspiration of blood was incised with a blade for further histopathologic analysis.
- In each example, please note the blood with foam coming out of the bronchi after cutting the edges of the lobes and the blood-filled lobules, suggesting aspiration of blood.
- A portion of the lung site suggesting aspiration of blood was incised with a blade and sent for histopathological analysis to confirm the presence of erythrocytes in the sites of gaseous exchange (alveoli, alveolar sac, alveolar ducts and respiratory bronchiole).

Atlas as supplementary material from the article "Agonic aspiration of blood: not useful as an animal-based indicator of electrical stunning ineffectiveness in pigs (*Sus domesticus*)"

For more information, please contact the corresponding author
(Mª Francisca Ferreira; franciscarcferreira@gmail.com)
2023

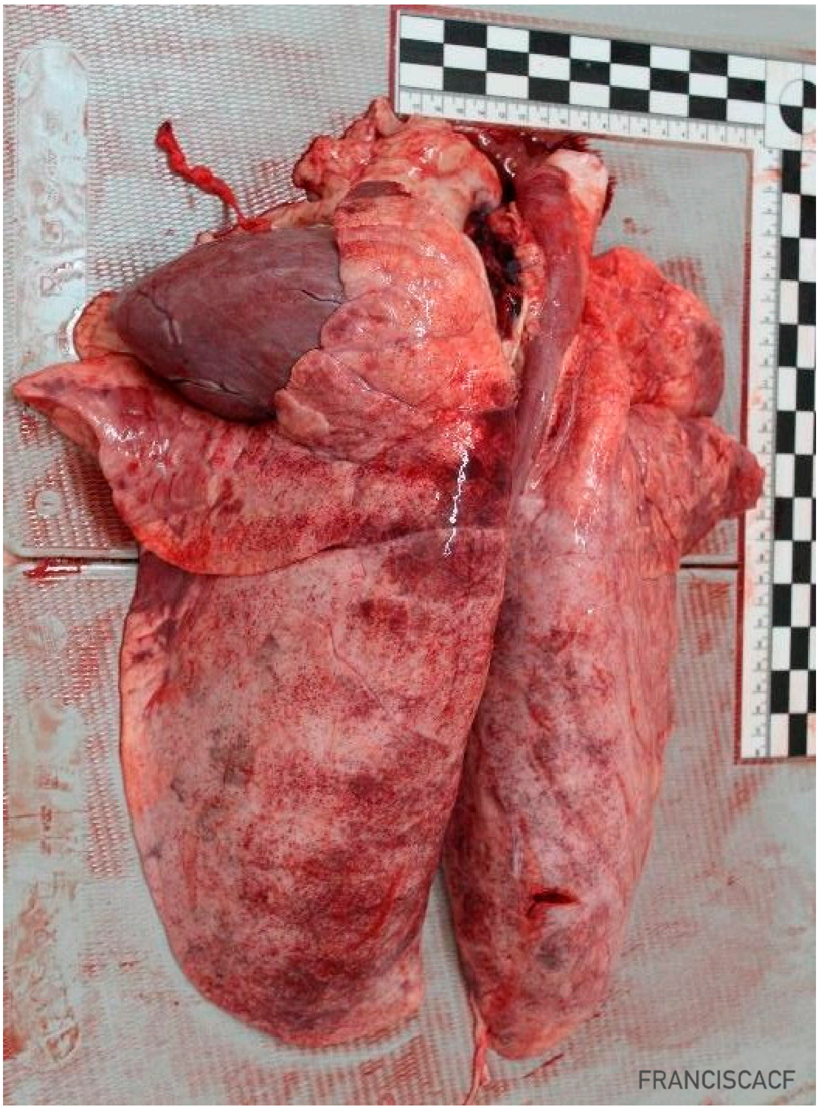
FRANCISCACF

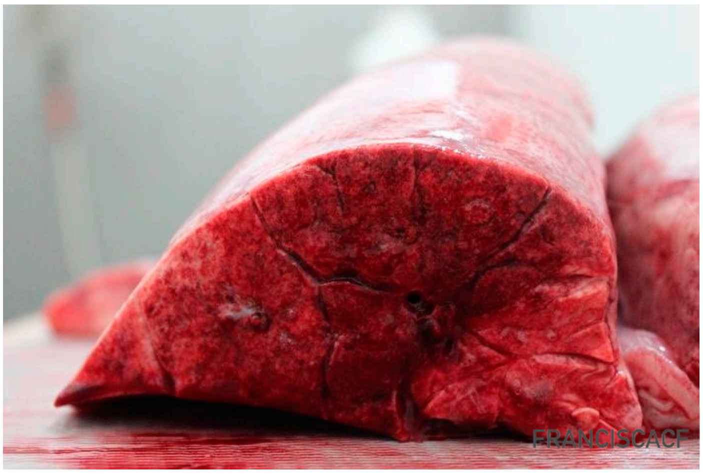
FRANCISCACF

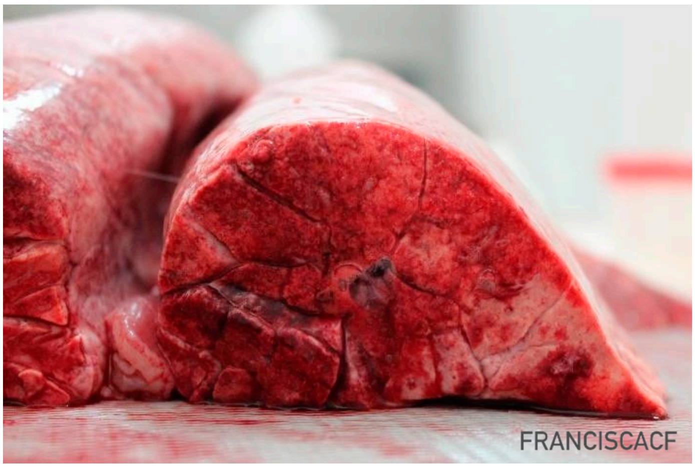
FRANCISCACF

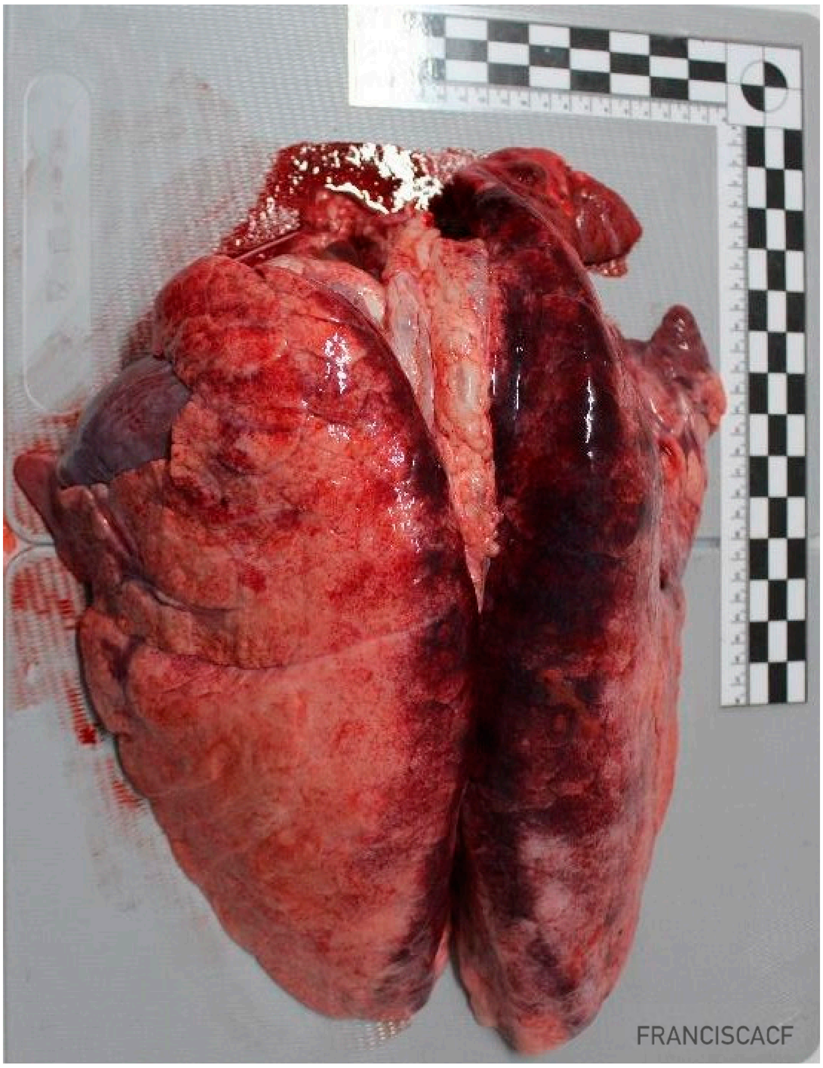
FRANCISCACF

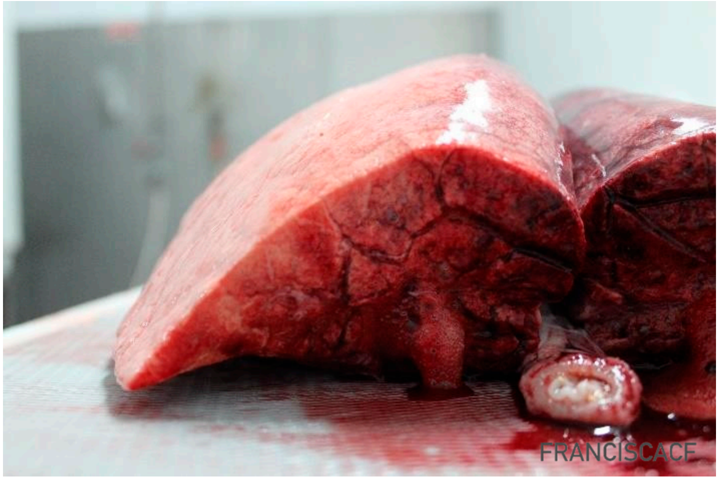
FRANCISCACF

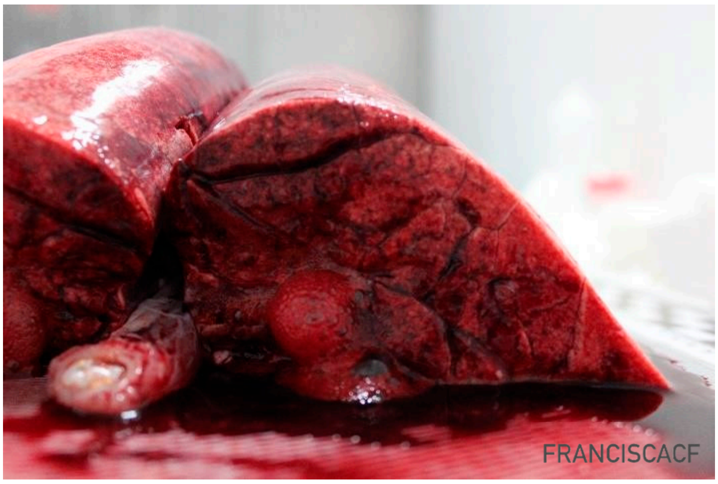
FRANCISCACF

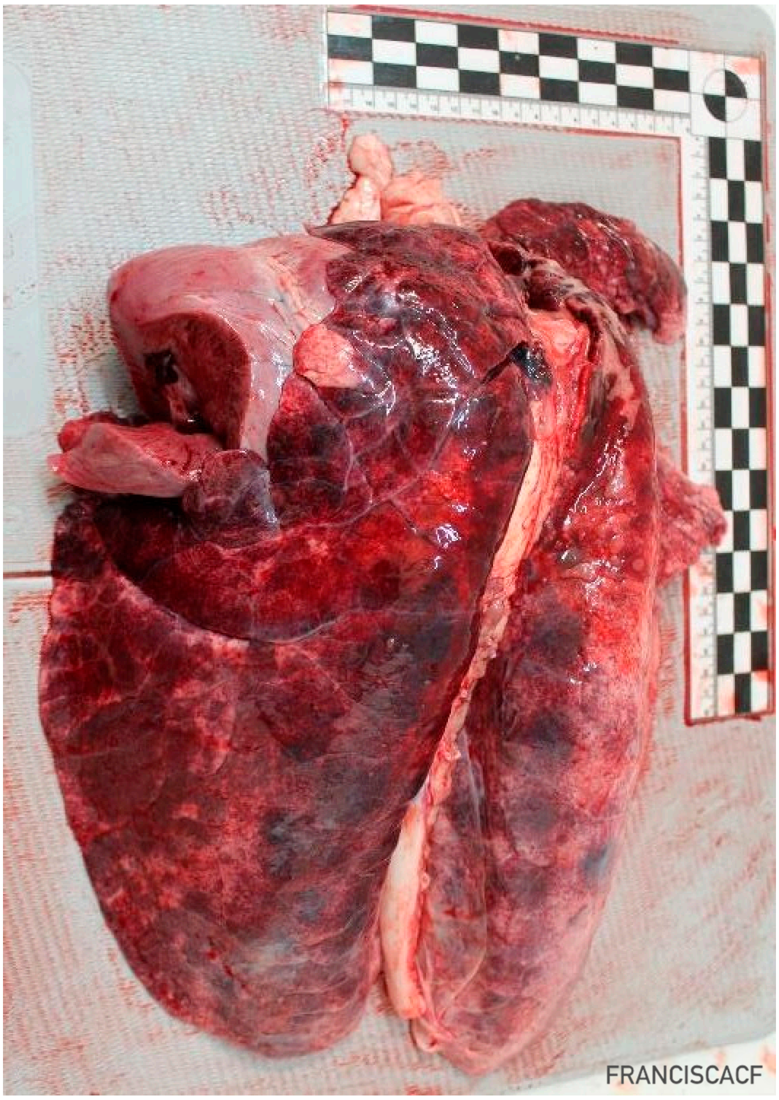
FRANCISCACF

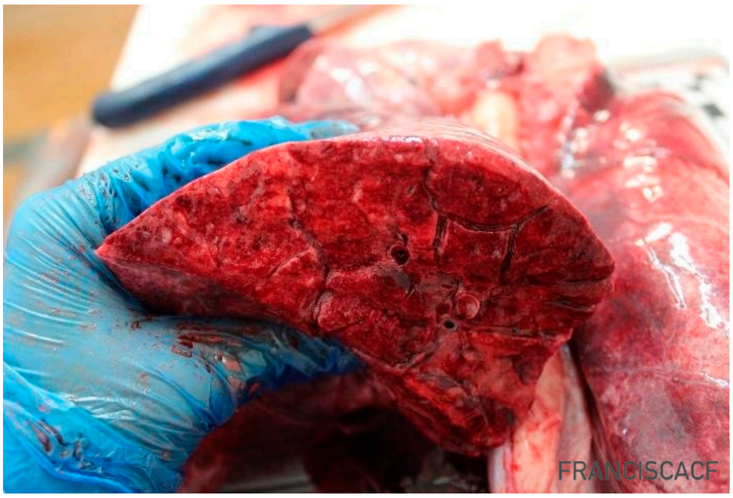
FRANCISCACF

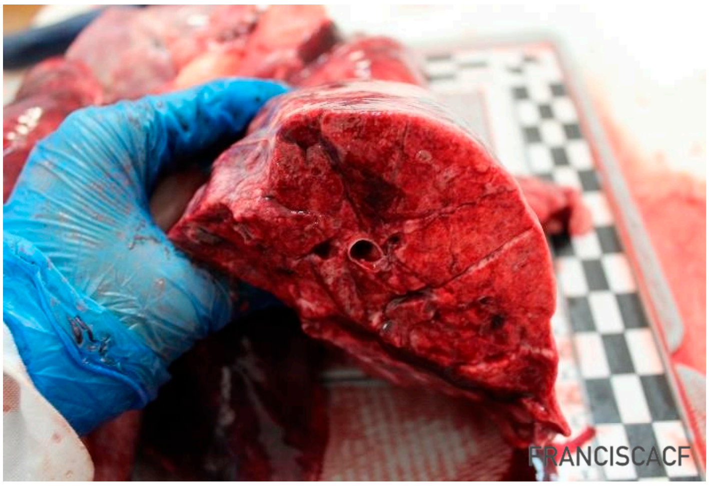
FRANCISCACF

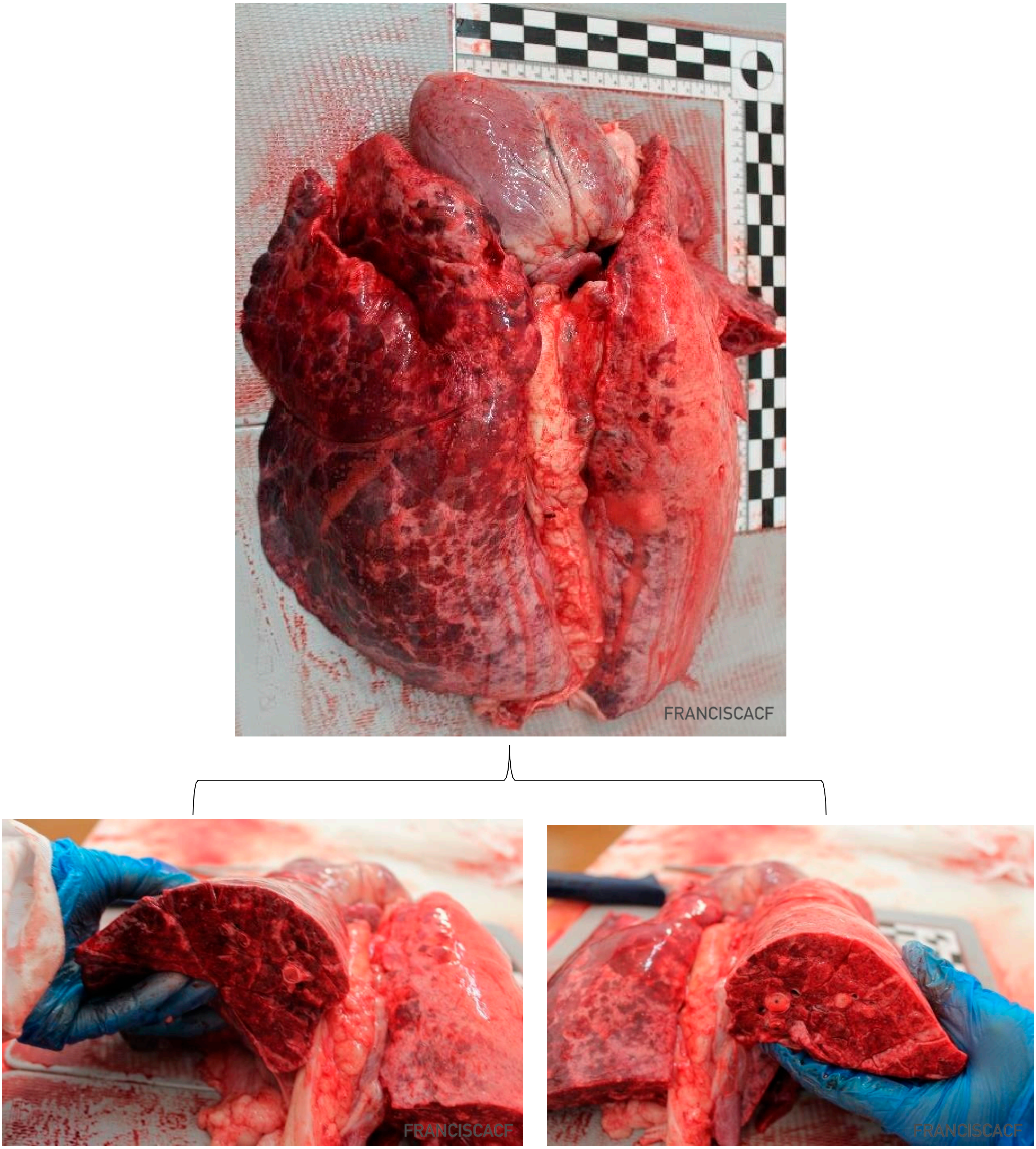
FRANCISCACF
FRANCISCACF
FRANCISCACF

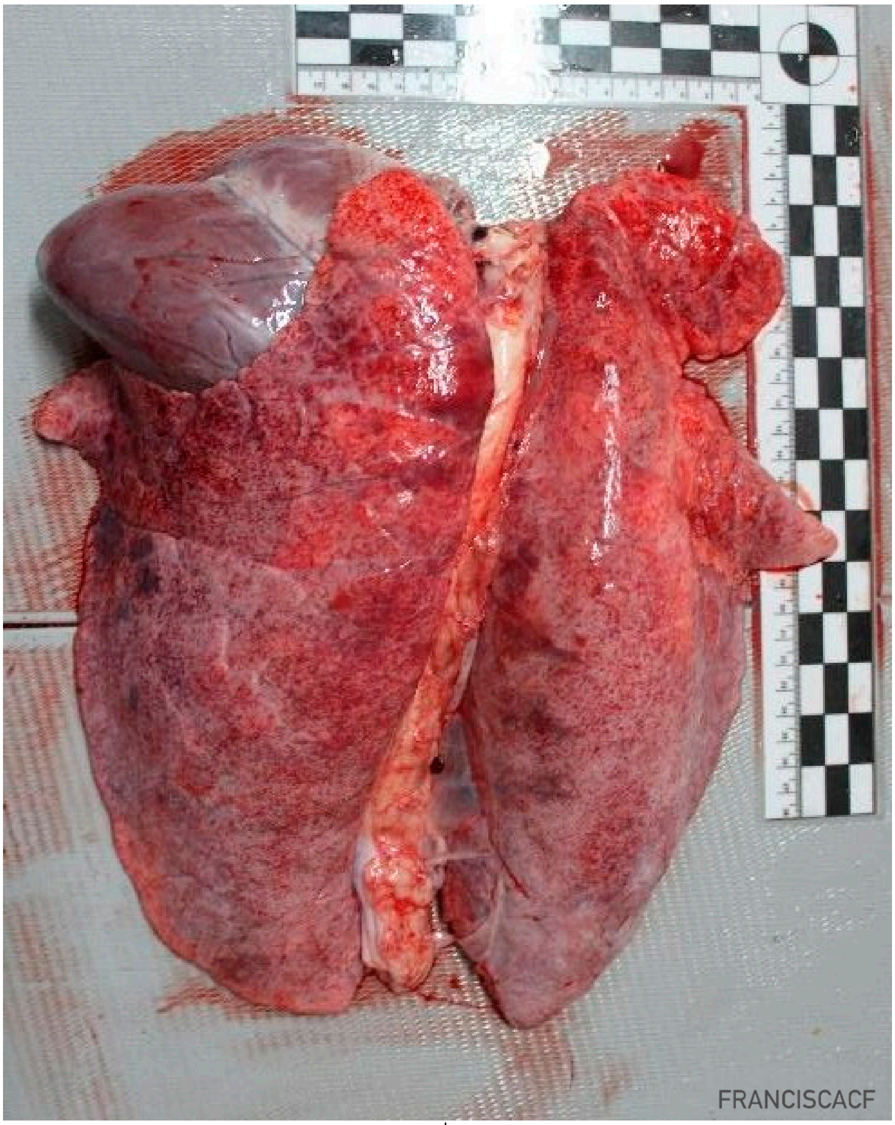
FRANCISCACF

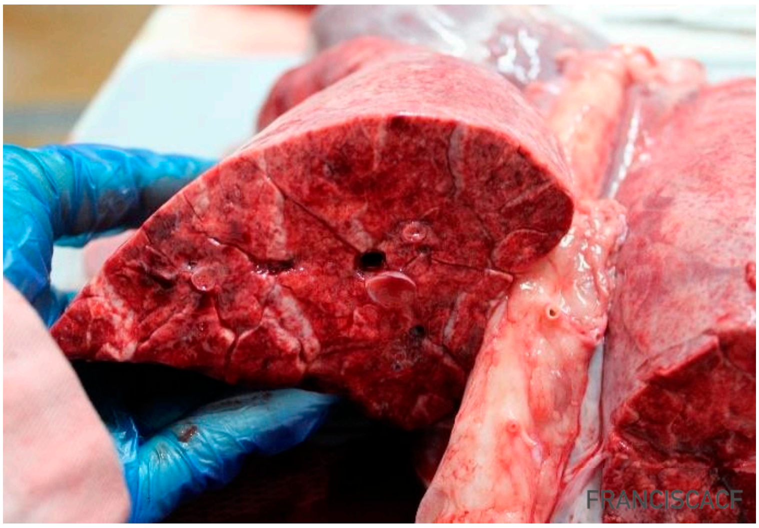
FRANCISCACF

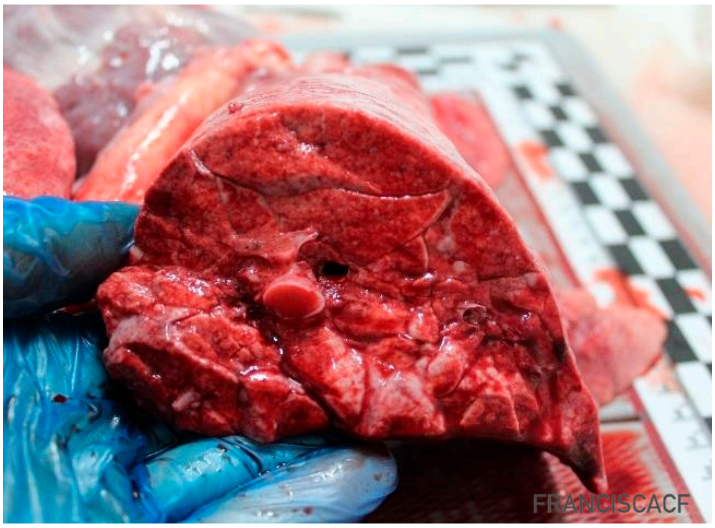
FRANCISCACF

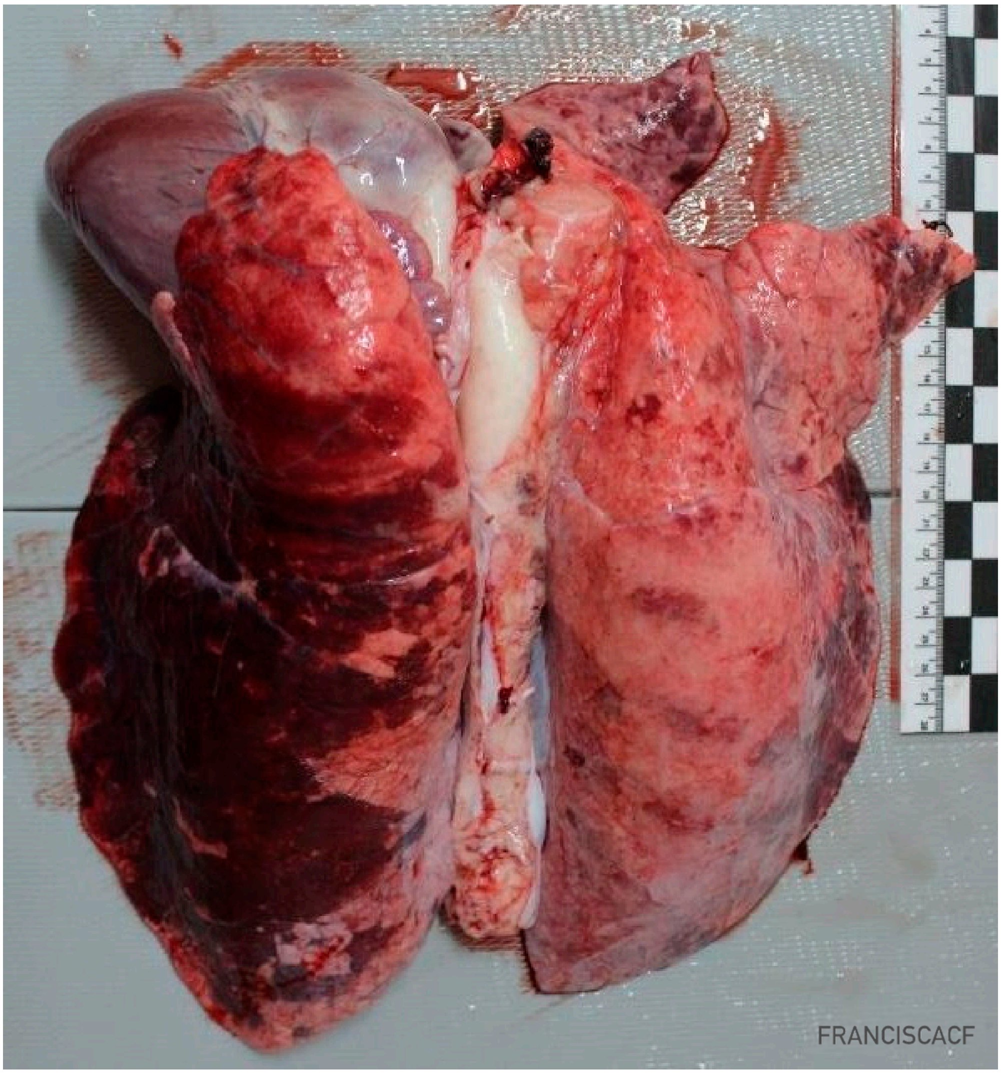
FRANCISCACF

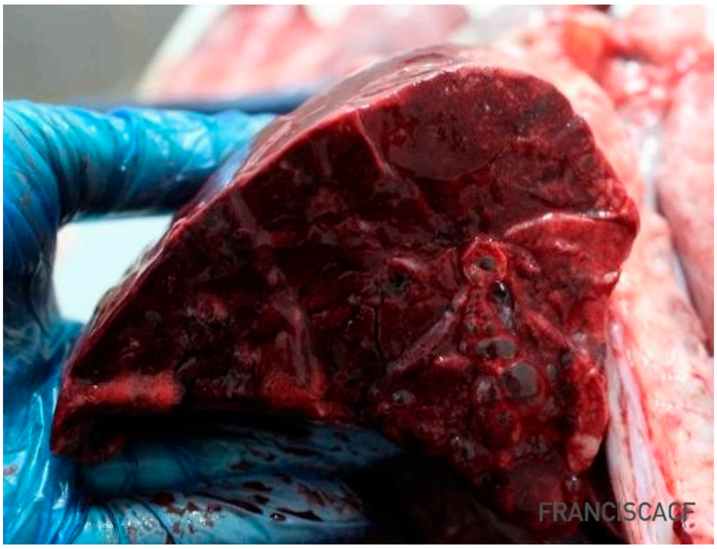
FRANCISCACF

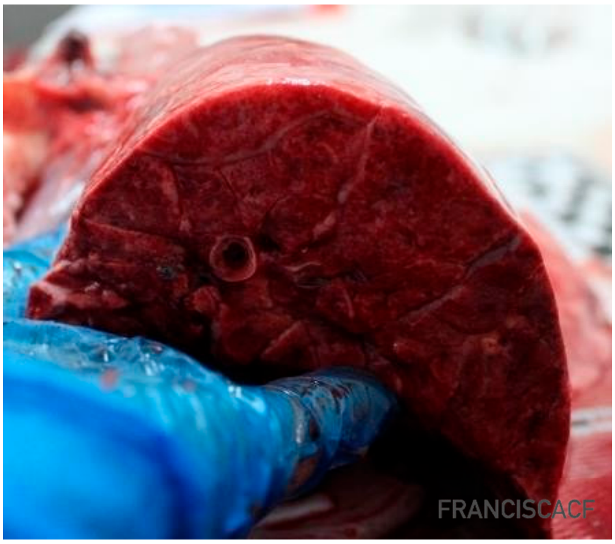
FRANCISCACF

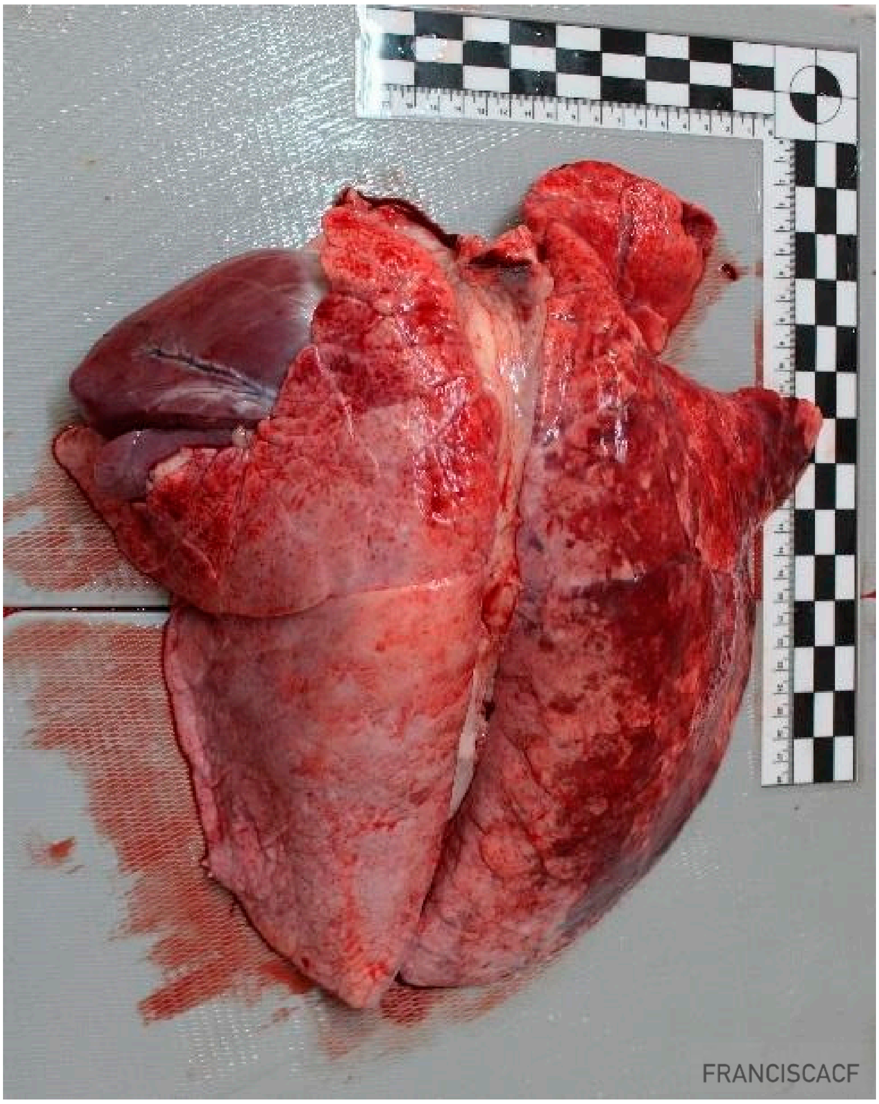
FRANCISCACF

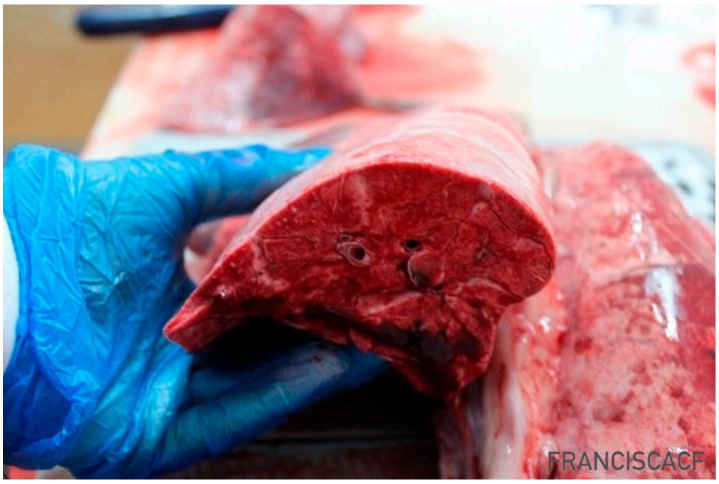
FRANCISCACF

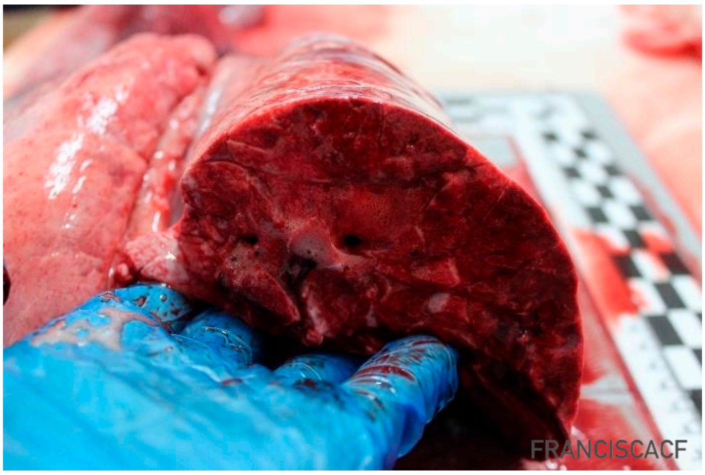
FRANCISCACF

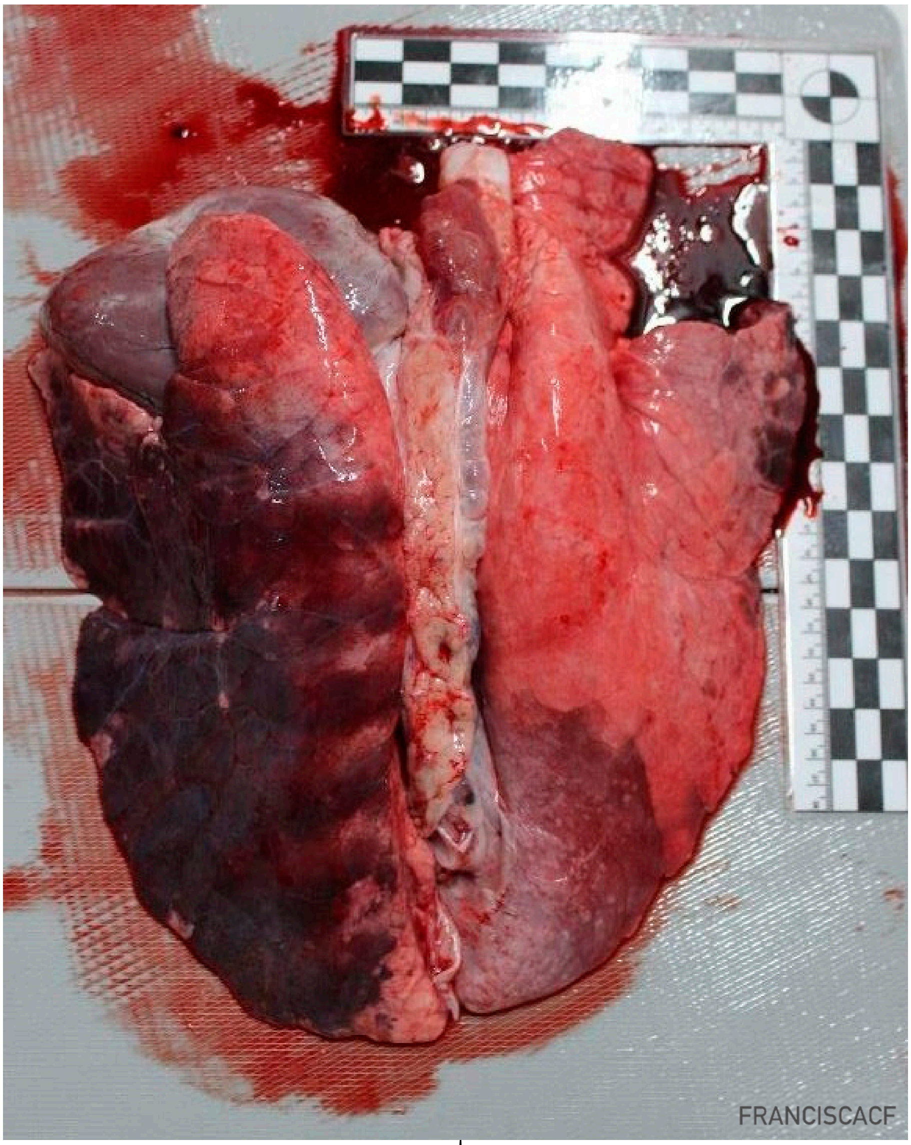
FRANCISCACF

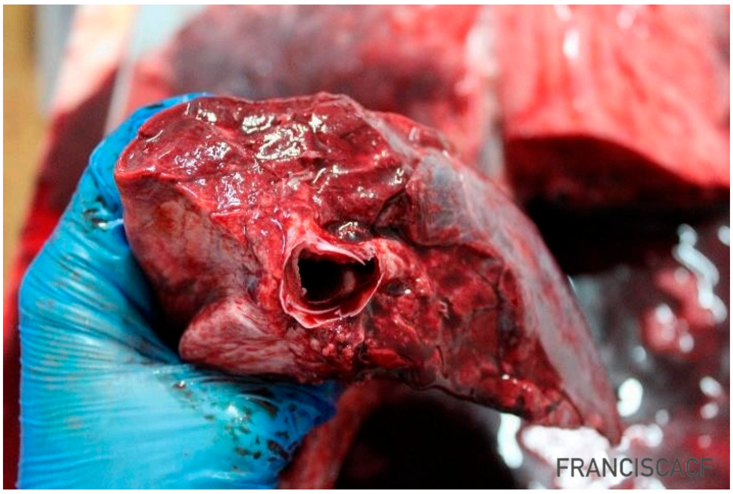
FRANCISCACF

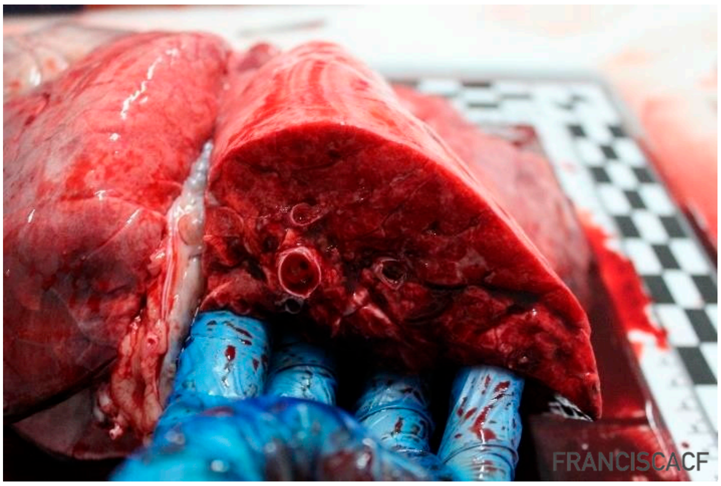
FRANCISCACF

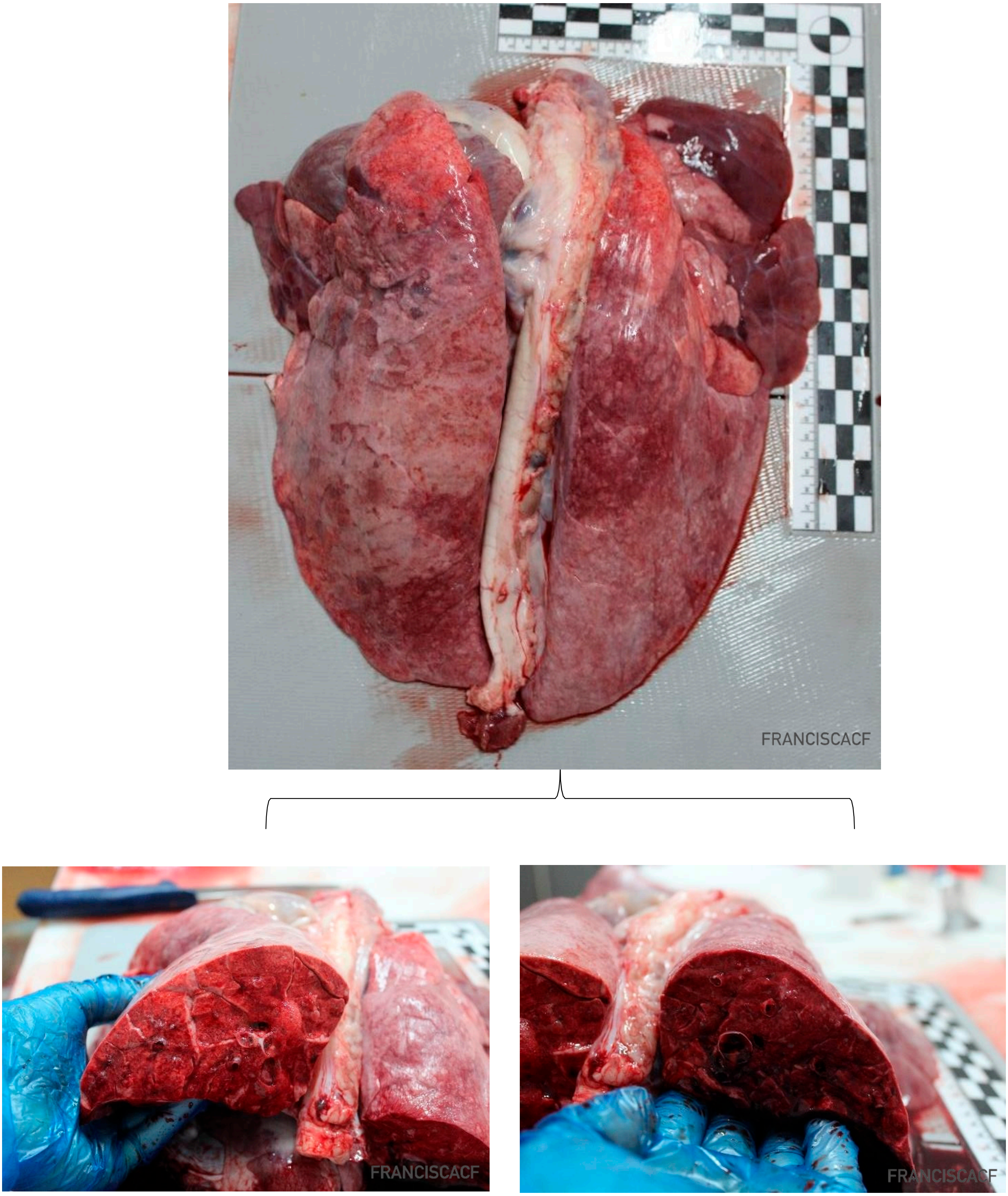
FRANCISCACF
FRANCISCACF

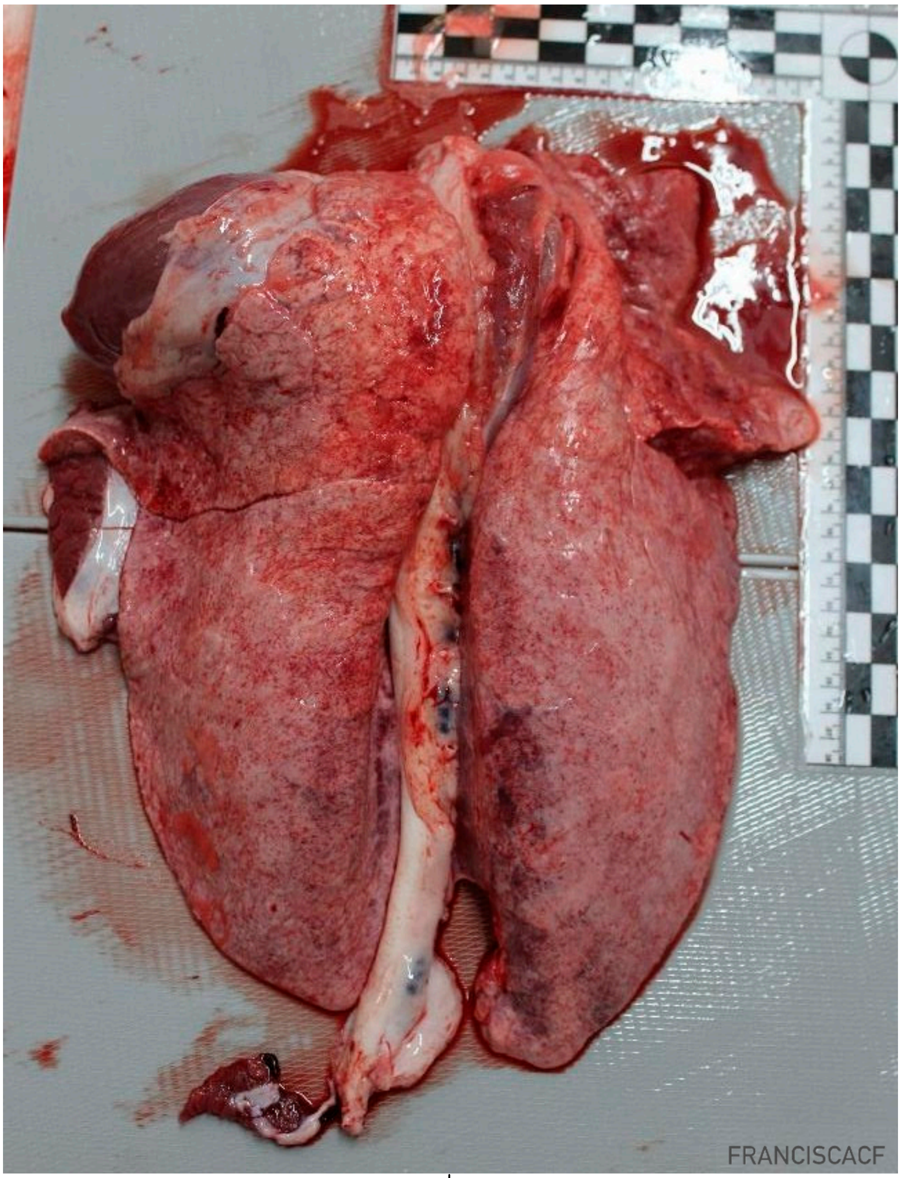
FRANCISCACF

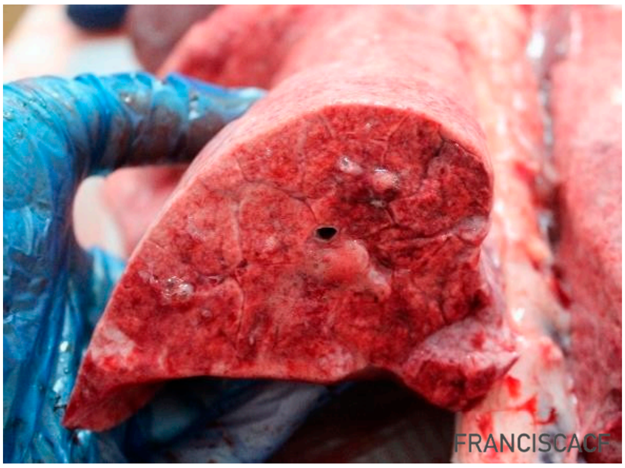
FRANCISCACF

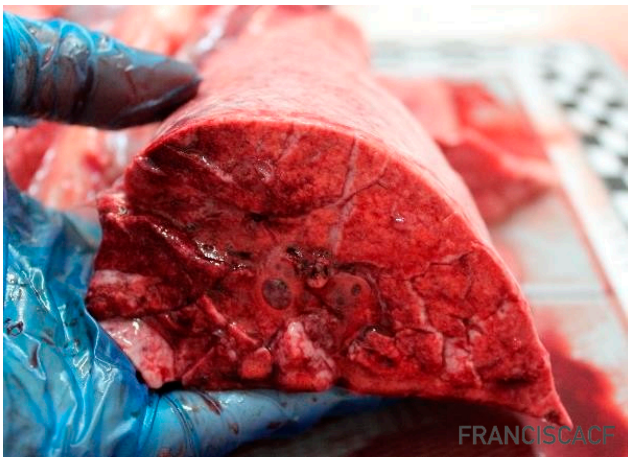
FRANCISCACF

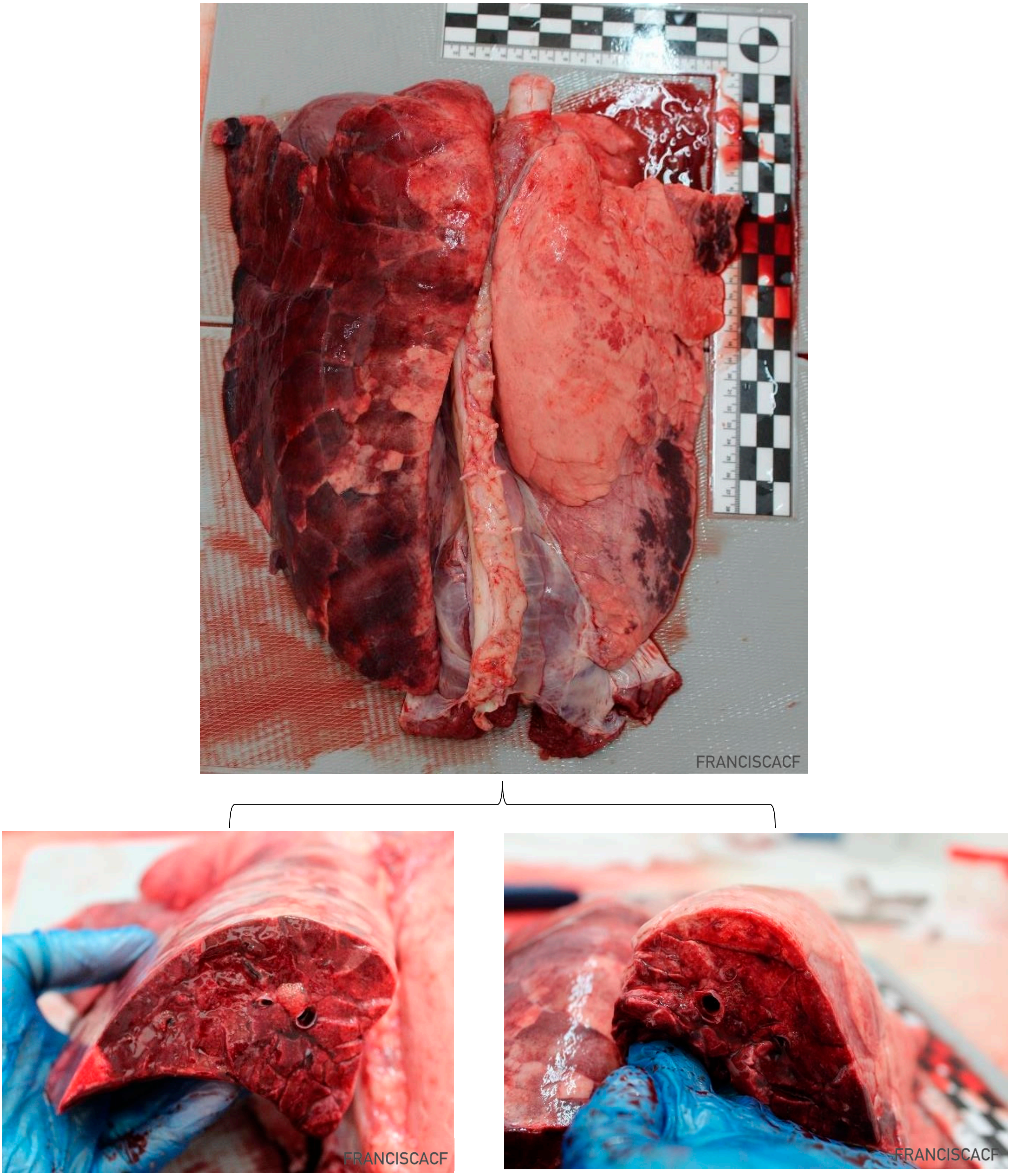
FRANCISCACF
FRANCISCACF
FRANCISCACF

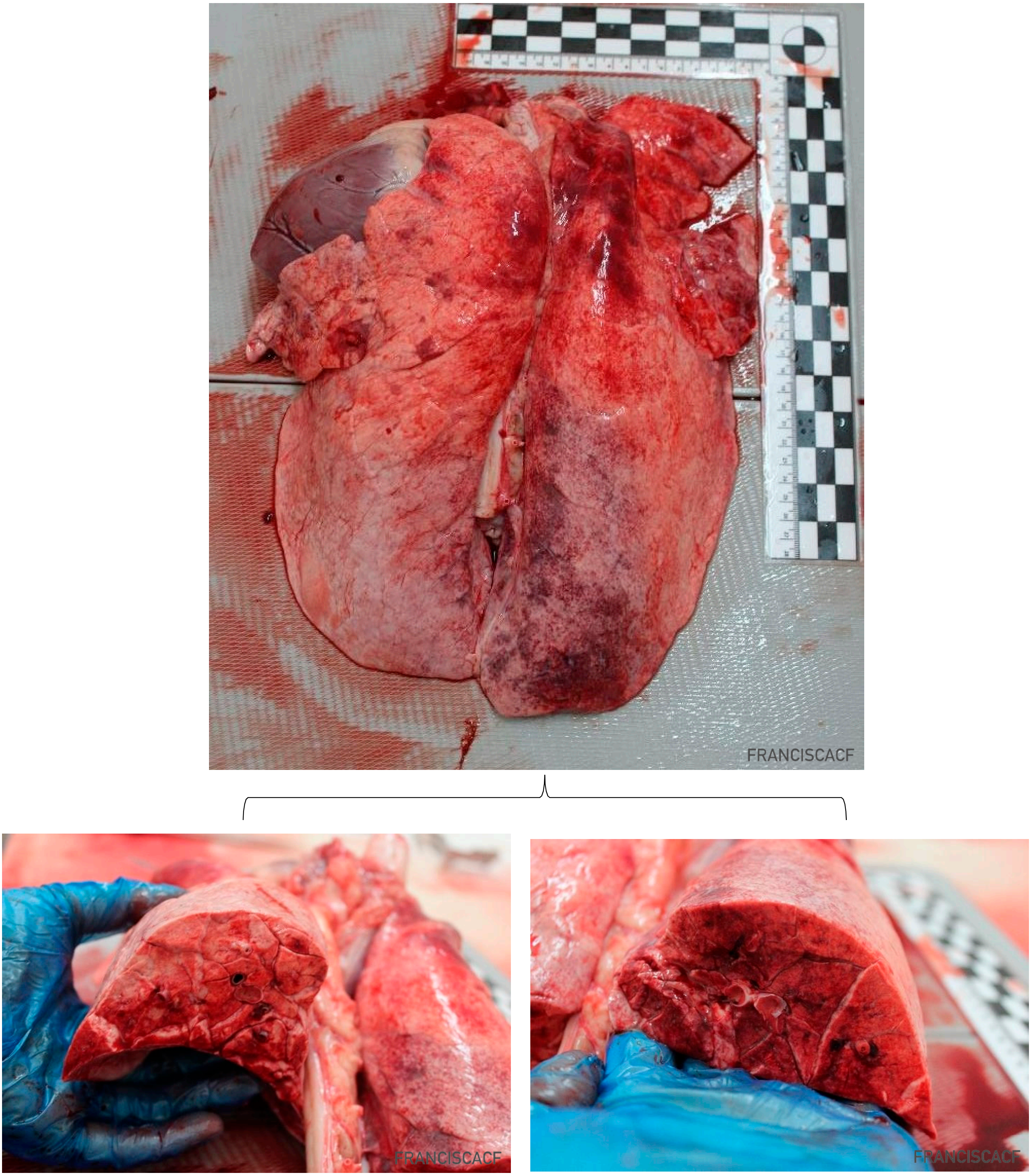
FRANCISCACF
FRANCISCACF
FRANCISCACF

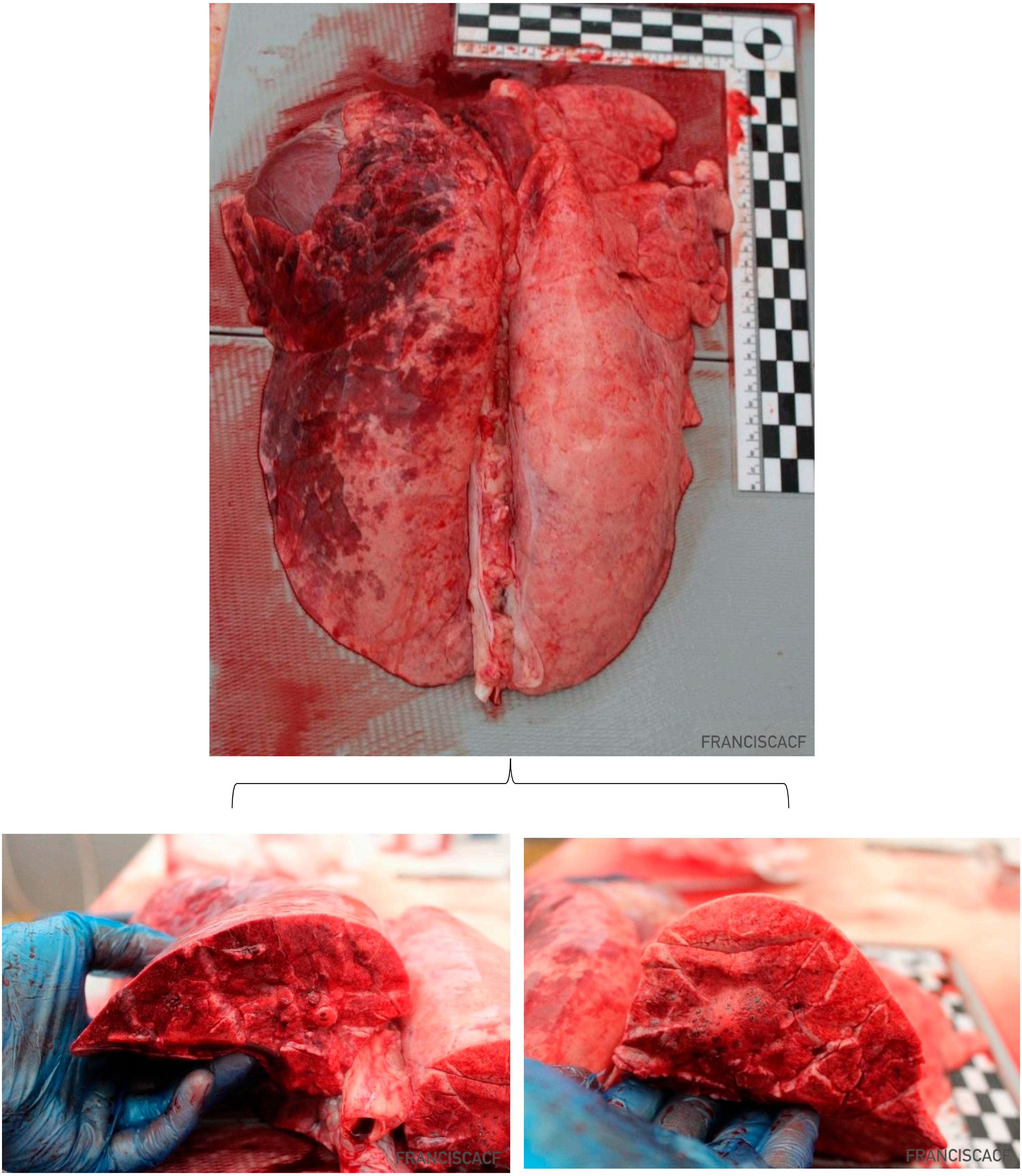
FRANCISCACF
